# SMRT-based mitochondrial genome of the edible mushroom *Morchella conica*

**DOI:** 10.1080/23802359.2020.1810160

**Published:** 2020-08-25

**Authors:** Wei Li, Fen Zhang, Li-zhi Gao

**Affiliations:** a Institution of Genomics and Bioinformatics, South China Agricultural University, Guangzhou, China; b Plant Germplasm and Genomics Center, Germplasm Bank of Wild Species in Southwestern China, Kunming Institute of Botany, Chinese Academy of Sciences, Kunming, China

**Keywords:** *Morchella conica*, mushroom, mitochondrial genome, PacBio, evolution

## Abstract

*Morchella conica* Pers. is a highly-prized mushroom for its edible and medical values. In this study, we determined the complete mitochondrial genome of *M. conica* combining both PacBio and Illumina sequencing technologies. The complete mitochondrial genome is 280,763 bp in length with a GC content of 39.88%. We identified a total of 14 core conserved protein-coding genes, 127 non-conserved open reading frames (ncORFs) and 30 tRNA genes in the *M. conica* mitogenome. However, no large or small rRNA subunits (rnl or rns) were identified in this mitogenome. In addition, we detected two mitochondrial RNase P (rnpB) genes and one ribosomal protein genes (rps3). Phylogenetic analysis was performed among *M. conica* and 18 other representative fungi from Ascomycota, Basidiomycota and Mucoromycota. Our results showed that *M. conica* was most closely related to *M. importuna*. The availability of the *M. conica* mitochondrial genome will form the basis of genetic breeding program and enhance our understanding of the evolution of this species.

Wild mushrooms have increasingly become an important part of human diet for centuries owing to its pleasant taste and nutritional value (Kalač 2009), among which morels were the most highly-prized for their immense economic and scientific values, widely consumed as food and medicine (Elmastas et al. 2007; Gençcelep et al. 2009; Wong and Chye 2009). *Morchella conica* Pers. is an edible mushroom belonging to the Ascomycota phylum (Masaphy and Zabari 2013). However, large-scale cultivation of *M. conica* has not been successful, and excessive harvesting without replenishment has led to a reduced effective population size, resulting in a global shortage and soaring market prices (Turkoglu et al. 2006). In this study, we assembled and annotated the complete mitochondrial genome of *M. conica* to provide genetic information for the future breeding programs.

The *M. conica* sample was collected from Shizong (103.99E, 24.83 N), Yunnan Province, China. Fresh fruit bodies were harvested and immediately frozen in liquid nitrogen after collection. Genomic DNA was extracted using a modified CTAB method (Porebski et al. 1997). One paired-end library was constructed following the Illumina’s instructions and sequenced on Illumina Hiseq2000 platform. For PacBio sequencing, a 40-kb SMRTbell DNA library was prepared and sequenced on Sequel II platform. The PacBio long reads were then assembled using CANU v1.6 (Koren et al. 2017). The assembled contigs were further polished using the paired-end Illumina reads with Pilon v1.22 (Walker et al. 2014). The mitochondrial genome of *M. importuna* (NC_045397) (Liu et al. 2020) was used as the reference to extract the mitogenome contigs using blast searches. One contig representing the complete mitochondrial genome was identified based on the sequence similarity. The mitochondrial genome of *M. conica* was annotated using MFannot webserver (http://megasun.bch.umontreal.ca/cgi-bin/mfannot/mfannotInterface.pl). The whole mitogenome sequence data reported here has been deposited in the Genome Warehouse in National Genomics Data Center (BIG Data Center Members 2020), Beijing Institute of Genomics (China National Center for Bioinformation), the Chinese Academy of Sciences, under accession number GWHANVR01000000 that is publicly accessible at https://bigd.big.ac.cn/gwh.

The mitochondrial genome of *M. conica* was 280,763 bp in length with a GC content of 39.88%. Fourteen conserved protein-coding genes encoded 3 ATP synthases (atp6, apt8 and apt9), 3 cytochrome oxidases (cox1, cox2 and cox3), apocytochrome b (cob) and 7 subunits of NAD dehydrogenase (nad1, nad2, nad3, nad4, nad5, nad6 and nad4L). Two mitochondrial RNase P (rnpB) genes and one ribosomal protein genes (rps3) were identified in the mitochondrial genome. Besides, 127 non-conserved open reading frames (ncORFs) were identified to be uniformly dispersed in the *M. conica* mitogenome. We also detected 30 tRNA genes, but no rRNA genes were found in the genome.

To investigate the phylogenetic position of *M. conica* we aligned 14 conserved protein-coding genes using MAFFT v7.305 (Katoh and Standley 2013). A maximum likelihood (ML) tree was constructed using Mega 6.0 (Tamura et al. 2013) with 1,000 bootstrap replicates. Our results showed that *M. conica* is closely related to *M. importuna* in the genus *Morchella* (Figure 1).

**Figure 1.**
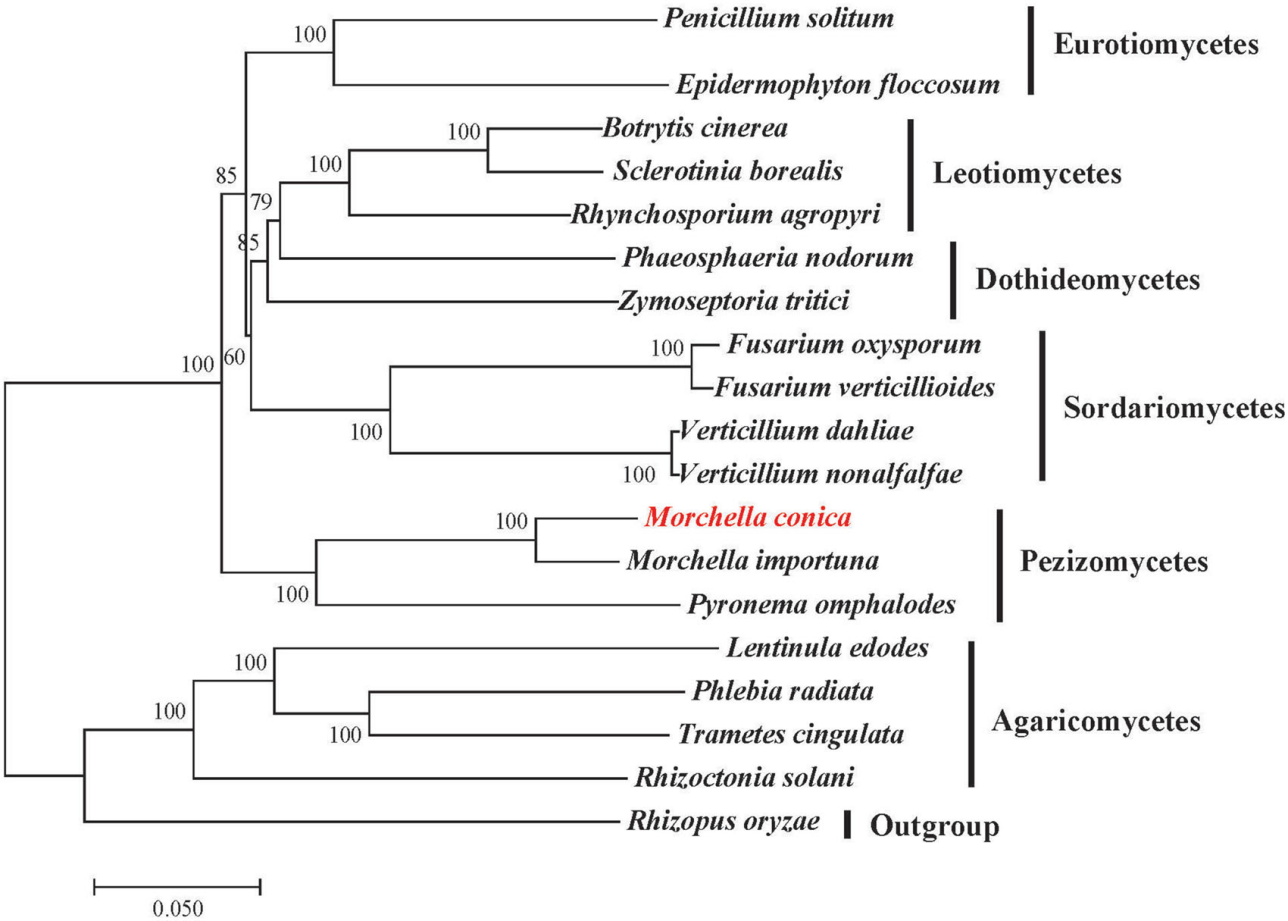
Maximum-likelihood (ML) phylogenetic tree of *M. conica* and related fungal species. The ML-tree is based on 14 conserved core mitochondrial proteins. Bootstraps values (1,000 replicates) are shown at the nodes. All the sequences are currently available in the GenBank database: *Botrytis cinerea* (KC832409), *Epidermophyton floccosum* (NC_007394), *Fusarium oxysporum* (NC_017930), *F. verticillioides* (NC_016687), *Lentinula edodes* (NC_018365), *M. importuna* (NC_045397), *Zymoseptoria tritici* (NC_010222), *Penicillium solitum* (NC_016187), *Phaeosphaeria nodorum* (NC_009746), *Phlebia radiata* (NC_020148), *Pyronema omphalodes* (NC_029745), *Rhizoctonia solani* (NC_021436), *Rhynchosporium agropyri* (NC_023125), *Sclerotinia borealis* (NC_025200), *Trametes cingulata* (NC_013933), *Verticillium dahliae* (NC_008248) and *V. nonalfalfae* (NC_029238). *Rhizopus oryzae* (NC_006836) was served as an outgroup.

## Data Availability

The data that support the findings of this study are openly available in the Genome Warehouse Database at https://bigd.big.ac.cn/gwh, accession number GWHANVR01000000.
